# Procalcitonin in Preterm Neonates: A Different Threshold and Prolonged Interpretation

**DOI:** 10.3389/fped.2021.623043

**Published:** 2021-05-17

**Authors:** Blandine Bianco, Bérengère François-Garret, Marine Butin, Cyril Dalmasso, Florence Casagrande, Mostafa Mokhtari, Sergio Eleni Dit Trolli

**Affiliations:** ^1^Neonatal Intensive Care Unit, CHU de Nice, Archet 2 Hospital, Nice, France; ^2^Neonatal Intensive Care Unit, Hospices Civils de Lyon, Hôpital Femme-Mère-Enfant, Bron, France; ^3^Laboratoire de Mathématiques et Modélisation d'Evry (LaMME), Université d'Evry Val d'Essonne, UMR CNRS 8071, Evry, France; ^4^Neonatal Intensive Care Unit, Assistance Publique-Hôpitaux de Paris, Kremlin-Bicêtre Hospital, University Paris Sud, Kremlin-Bicêtre, France

**Keywords:** procalcitonin, early-onset sepsis, preterm, biomarker, threshold

## Abstract

**Objectives:** To evaluate the positive threshold of PCT for neonates of <32 weeks of gestation for the diagnosis of early-onset sepsis and to determine if the level of PCT collected within 6 h of life could be used.

**Design:** Retrospective and bicentric study from May 2016 to April 2018.

**Setting:** Two groups were established, neonates evaluated for PCT at birth (CordPCT) and within 6 h of life (delPCT).

**Patients:** Two hundred and sixty neonates of <32 weeks of gestation born in Nice and South Paris (Bicêtre) University Hospitals, had been evaluated for PCT level.

**Main Outcomes Measures:** The value of the PCT positive threshold was determined for the total population and each groups thanks ROC curves.

**Results:** The threshold level of PCT for the total population was 0.98 ng/mL. The threshold value of cordPCT group was 1.00 vs. 0.98 ng/mL for delPCT group. The area under the Receiver Operating Characteristics curve for PCT sampled in delPCT group was significantly higher than in cordPCT group (0.94 compared to 0.75).

**Conclusions:** The threshold level of PCT was higher in this cohort of neonates of <32 weeks of gestation compared to the value generally described for term neonates. The secondary sampling PCT level seems to be usable in screening algorithm for early-onset neonatal sepsis.

## Introduction

Early-onset neonatal sepsis (EOS) is defined as a systemic infection occurring within the first 72 h of life (1). The incidence of EOS among premature neonates <1,500 g is 8–26 for 1,000 births (2). Infection is responsible for the high morbidity and mortality for preterm infants (3), due to the immaturity of their innate immune system (4). Diagnosis is still difficult when considering the perinatal infection risk factors, such as non-specific clinical symptoms (5, 6) biomarkers of low sensitivity and blood cultures that are often negative (7).

The majority of extremely premature neonates [78.6% of neonates of <1,500 g and 87% of <1,000 g (8)] receive large spectrum antibiotic therapy immediately in postnatal, which is often justified within a context of maternal infection and non-specific or absent neonatal clinical symptoms (9). Antibiotic therapy has short-term consequences: delay in colonization of the intestinal microbiota, decrease in the diversity in favor of bacterial pathogens (*Enterobacter, Enterococcus* and *Streptococcus*) (10–12), increase in the risk of delayed bacterial infection, necrotizing enterocolitis and death (13). Long-term modifications to the intestinal microbiota result in an increased risk of developing diabetes, allergies (asthma, eczema) and chronic inflammatory bowel disease (14).

The prohormone procalcitonin (PCT) is more sensitive than C-reactive protein (CRP) and the leucocyte count in diagnosis of EOS (15–17). However, a few issues concerning PCT must be understood when interpreting the clinical relevance of the level: the physiological increase during the first few days of life (18–20) and the kinetic differences for preterm infants (21) [delayed plasmatic peak and prolonged return to the basal level (19, 22)]. The PCT level of the umbilical cord avoids the postnatal physiological increase. The threshold positive level for PCT of the umbilical cord is 0.6 ng/mL (17) and is the same for neonates, whatever the term of birth. Only a few studies have evaluated the level of PCT of the umbilical cord in premature neonates (21, 23) and only one regarding neonates of <28 weeks (24). Neonates <32 weeks represent a small percentage of babies included in these studies. The data are discordant concerning the possible effect of gestational age on PCT (22, 25, 26). Defining the positive threshold value for neonates of <32 weeks will improve the diagnosis of EOS in this population by better targeting newborns at risk and limiting unnecessary antibiotic therapy.

Most studies concentrate on the PCT of the umbilical cord due to its high post-natal physiological level. A physiologic increase in the PCT level occurs after the first 6 h of life (27). The aim of this work is also to assess PCT before the first 6 h of life during admission to the critical care unit and during intensive neonatal care.

The main objective of this study was to determine the positive threshold level of PCT in premature neonates <32 weeks. The secondary objective was to compare the level of PCT obtained during the first 6 h of life with the level of the umbilical cord at birth.

## Patients and Methods

This retrospective, observational and dicentric study was performed between 1 May 2016 and 30 April 2018 in the neonatal critical care units of the Nice and South Paris (Bicêtre) University Hospitals. Written or oral consent was not required. This study was registered with the ≪ Commission Nationale de l'Informatique et des Libertés (CNIL) ≫ under the methodology MR-003 and reference R026.

### Inclusion and Exclusion Criteria

All the premature neonates born in one of the two centers who presented infection risk factor(s) and sampled for PCT at birth or in the first 6 h of life were included in this study. Infection risk factors have been defined by the ≪ Haute Autorité de Santé (HAS) ≫ (28) and the ≪ Société Française de Néonatalogie (SFN) ≫ (29). Neonates without PCT evaluation, without infection risk factors, born outside of a hospital with a neonatal critical care unit or with malformations diagnosed antepartum or with chromosomic anomalies were not included. Neonates were excluded if the infectious status had not been determined due to incomplete clinical or biological data.

### PCT Laboratory Test

A sample of whole blood of 500 μL was obtained from an umbilical cord vein (from the cord or after introducing a catheter into the umbilical vein) or from a peripheral vein after introducing a peripheral venous line. Microvette^®^ 500 Hep-Li-Gel (SARSTEDT, Nümbrecht, Germany) sampling tubes containing lithium heparin and a separating gel were used. The PCT level was evaluated by sandwich immunoassay and electro chemiluminescence. The Nice University Hospital used a ADVIA Centaur CP (SIEMENS HEALTHINEERS, Erlangen, Germany) system with the ADVIA Centaur BRAHMS PCT reactive. The Bicêtre University Hospital used a COBAS^®^ 8000 (ROCHE DIAGNOSTICS, Bâle, Switerland) system with the Elecsys BRAHMS PCT reactive. Comparative tests for PCT using the different methods using the BRAHMS reactive were performed. The result of analysis of PCT with the different reactive were the same (correlation coefficient close to one).

### EOS Classification

The classification of the neonates was made retrospectively according to the infectious status (certain, probable or absent infection) by two neonatalogists blinded to the results of the PCT level. In the case of disagreement a third neonatalogist was consulted concerning the infectious status of the infant.

Infection was certain if a central sample was positive (blood culture and/or lumbar puncture). Infection was probable if the neonate had symptoms and/or had a level of CRP ≥ 10 mg/mL (30, 31), which may be associated to a positive gastric fluid culture and/or an anomaly in blood count and leukocytic formula (leukocytes < 5,000/mm3 or > 25,000/mm3 or > 25,000/mm3 or thrombocytopenia <150,000/mm3). A neonate is symptomatic if it presented with the following: hypothermia/hyperthermia, tachycardia/ bradycardia, arterial hypotension/poor perfusion, apnea, respiratory distress, lethargy, seizures or digestive intolerance. Premature neonates showing only respiratory and/or digestive symptoms due to frequent symptoms of respiratory distress and feeding difficulties during the 1st days of life were differentiated.

### Patient Classification

The threshold level of PCT was determined for the entier population (≪ allPCT ≫), and then for two distinct groups depending on the time of sampling for PCT analysis: at birth (≪ cordPCT ≫) and after birth during admission into neonatal intensive care (≪ delPCT ≫).

### Statistical Analysis

A descriptive analysis of the characteristics of the population expressed as frequencies (percentages) for quantitive variables and medians (minimal-maximal values) for quantitive variables is provided. The non-parametric Wilcoxon and the Fisher exact tests were used to compare the characteristics of the population and the level of PCT (patients infected and not infected in the groups ≪ allPCT ≫, ≪ cordPCT ≫ and ≪ delPCT ≫). The Receiver Operating Characteristics (ROC) curves were calculated to determine the positive threshold value for the different populations and their sensitivity, specificity, and positive and negative predictive values. The areas under the curve (AUC) were compared using the method of DeLong et al. (32). The level of significance was set at 5% (a result with a *p*-value below 5% was considered as statistically significative). The statistics and the ROC curves were obtained using R software version 3.6.0.

## Results

### Description of the Population

**Figure 1:**
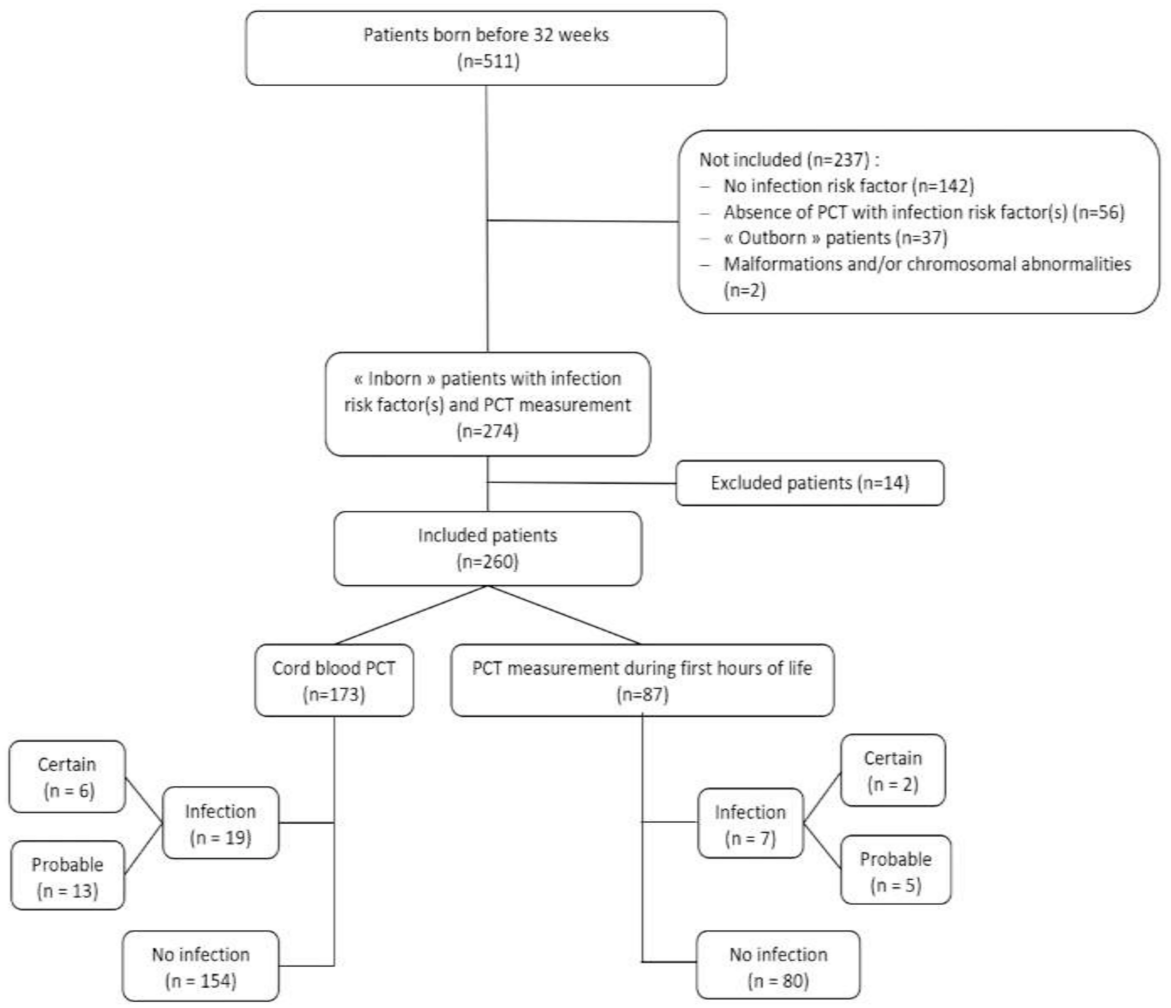
Study flow diagram. PCT, procalcitonin.

Five hundred and eleven premature neonates <32 weeks were taken into care in type III centers, Nice and Paris (Bicêtre) University Hospitals, between 1 May 2016 and 30 April 2018. Samples for PCT were obtained from 273 neonates presenting with infection risk factor(s), 13 were excluded because of the lack of data concerning the infectious status (Figure 1). Two hundred and sixty neonates were included into the statistical analysis; 173 samples for PCT were obtained at birth (group ≪ cordPCT ≫) and 87 in the first 6 h of life (groupe ≪ delPCT ≫). Among the 260 neonates, 26 had an infection (18 probable and eight certain infections). The clinical characteristics of the included neonates are presented in Table 1.

**Table 1:** Baseline characteristics of patients and their mothers in the “allPCT,” “cordPCT” and “delPCT” groups (the statistical analysis compared the “cordPCT” and “delPCT” groups).

	**≪ allPCT ≫ group (*n* = 260)**	**≪ cordPCT ≫ group (*n* = 173)**	**≪ delPCT ≫ group (*n* = 87)**	***p*-value**
Gestational age (weeks)				0.06
Median (IQR)	28.9 (27; 30)	29.1 (27; 31)	28.6 (27.1; 29.9)	
Min; max	23.9; 31.9	24.4; 31.9	23.9; 31.9	
Male sex, No. (%)	146 (56.2)	97 (56.1)	49 (56.3)	1.00
Birth weight (g)				**0.04**
Median (IQR)	1200 (970; 1450)	1240 (970; 1470)	1130 (955; 1355)	
Min; max	520; 2240	540; 2240	520; 1820	
Antenatal glucocorticoids exposure, No. (%)	245 (94.2)	165 (95.4)	80 (92)	0.27
Magnesium sulfate, No. (%)	210 (80.8)	145 (83.8)	65 (74.7)	0.13
Premature rupture of membranes, N (%)	170 (65.4)	119 (68.8)	51 (58.6)	0.13
Prolonged rupture of membranes (≥ 12 h), No. (%)	143 (55)	102 (59)	41 (47.1)	0.13
Cesarean, No. (%)	105 (40.4)	61 (35.3)	44 (50.6)	**0.02**
Clinical chorioamnionitis, No. (%)	128 (49.2)	86 (49.7)	42 (48.3)	0.90
Prenatal antibiotic exposure, No. (%)	203 (78.1)	147 (85)	56 (64.4)	**<0.001**
Pre eclampsia, No. (%)	3 (1.2)	2 (1.2)	1 (1.1)	1.00
Gestational diabetes, No. (%)	32 (12.3)	21 (12.1)	11 (12.6)	1.00
Intrauterine growth retardation, No. (%)	5 (1.9)	2 (1.2)	3 (3.4)	0.34
Threat of preterm birth, No. (%)	221 (85)	150 (86.7)	71 (81.6)	0.28
Fetal rhythm abnormalities, No. (%)	96 (36.9)	64 (37)	32 (36.8)	1.00
Apgar at 5 min < 7, No. (%)	37 (14.2)	21 (12.1)	16 (18.4)	0.19
pH at birth				0.79
Median (IQR)	7.32 (7.28; 7.32)	7.32 (7.28; 7.37)	7.32 (7.28; 7.38)	
Min; max	6.88; 7.54	7.05; 7.54	6.88; 7.49	
Multiple pregnancy, No. (%)	96 (36.9)	58 (33.5)	38 (43.7)	0.10
Positive vaginal swab, No. (%)	137 (52.7)	94 (54.3)	43 (49.4)	0.41
Infection, No. (%)	26 (10)	19 (11)	7 (8)	0.52
Certain, No. (%)	8 (3.1)	6 (3.5)	2 (2.3)	1.00
Probable, No. (%)	18 (6.9)	13 (7.5)	5 (5.7)	1.00
Postnatal antibiotic exposure, No. (%)	195 (75)	135 (78)	60 (69)	0.13
Death, No. (%)	41 (15.8)	23 (13.3)	18 (20.7)	0.11
Due to early-onset sepsis, No. (%)	9 (3.5)	6 (3.5)	3 (3.4)	0.48

*IQR, Interquar tile range*.

*Bold values indicates statistically significant difference*.

### PCT and EOS (“allPCT” Group)

The median PCT of infected neonates was significantly higher compared to non-infected neonates (3.45 vs. 0.33 ng/mL, *p* < 0.001). *Escherichia coli* was the pathogen detected in the majority of the certain and probable infections (Table 2).

**Table 2:** Pathogens of certain and probable infections in the ≪ allPCT ≫, ≪ cordPCT ≫ and ≪ delPCT ≫ groups.

	**≪ allPCT ≫ group (*n* = 260)**	**≪ cordPCT ≫ group (*n* = 173)**	**≪ delPCT ≫ group (*n* = 87)**
Infection, No. (%)	26 (10.0)	19 (11.0)	7 (8.0)
Certain infection, No. (%)	8 (3.1)	6 (3.5)	2 (2.3)
*Escherichia coli*, No.	3	2	1
*Capnocytophaga sputigena*, No.	1	1	0
*Corynebacterium aurimucosum*, No.	1	0	1
*Enterobacter cloacae*, No.	1	1	0
*Enterococcus faecalis*, No.	1	1	0
*Streptococcus mitis*, No.	1	1	0
Probable infection, No. (%)	18 (6.9)	13 (7.5)	5 (5.7)
*Escherichia coli*, No.	10	6	4
*Streptococcus agalactiae*, No.	1	1	0
*Streptocuccus mitis*, No.	1	1	0
Others *Streptococcus*, No.	1	1	0
*Candida albicans*, No.	1	1	0
*Citrobacter koseri*, No.	1	1	0
*Enterococcus faecalis*, No.	1	1	0
*Negative*, No.	2	1	1

*Bacteria of certain infections were detected in hemocultures or cerebrospinal fluid cultures. Pathogens of probable infections were detected in newborns (gastric fluid) and/or in their mothers (vaginal swab, placenta, amniotic fluid, endocol, urine)*.

Analysis of the ROC curve gave a positive threshold value of PCT of 0.98 ng/mL; sensitivity of 0.65 (IC 95% 0.50–0.85), specificity of 0.90 (IC 95% 0.57–0.97), predictive positive value of 0.42 (IC 95% 0.17–0.67) and negative predictive value of 0.96 (IC 95% 0.94–0.98) (Table 3).

**Table 3:** Diagnostic values for procalcitonin (PCT) in the ≪ allPCT ≫, ≪ cordPCT ≫ and ≪ delPCT ≫ groups.

**Procalcitonin**	**≪ allPCT ≫ group**	**≪ cordPCT ≫ group**	**≪ delPCT ≫ group**
Threshold (ng/mL)	0.98	1.00	0.98
Sensitivity (95% CI)	0.65 (0.5–0.85)	0.63 (0.47–0.84)	0.86 (0.71–1)
Specificity (95% CI)	0.90 (0.57–0.97)	0.89 (0.5–0.98)	0.93 (0.66–1)
Positive predictive value (95% CI)	0.42 (0.17–0.67)	0.42 (0.15–0.82)	0.50 (0.21–1)
Negative predictive value (95% CI)	0.96 (0.94–0.98)	0.95 (0.93–0.98)	0.99 (0.97–1)

### Cord Blood PCT and EOS (“cordPCT” Group)

Among the 260 samples obtained for PCT evaluation, 173 were from umbilical cord blood. Nineteen neonates in group ≪ cordPCT ≫ presented a probable or certain EOS. The average PCT of the umbilical cord of infected neonates was significantly higher compared to non-infected neonates (3.02 vs. 0.32 ng/mL, *p* < 0.001).

The threshold positive PCT level of the umbilical cord obtained from the ROC curve was 1 ng/mL; sensitivity of 0.63 (IC 95% 0.47–0.84), specificity of 0.89 (IC 95% 0.5–0.98), predictive positive value of 0.42 (IC 95% 0.15–0.82) and predictive negative value of 0.95 (IC 95% 0.93–0.98) (Table 3).

### PCT Level During the 1st h of Life and EOS (“delPCT” Group)

The 87 blood samples of “delPCT” group were obtained up to 3 h and 39 min after birth. The median hour of sampling was 60 min (20–219). Seven neonates presented a probable or certain EOS. The median PCT level in the 1st h of life of the infected neonate was significantly higher compared to non-infected neonates (9.09 vs. 0.36 ng/mL, *p* < 0.001).

The threshold value determined from the ROC curve was 0.98 ng/mL when samples were obtained in the first 6 h of life; sensitivity of 0.86 (IC 95% 0.71–1.00), specificity of 0.93 (IC 95% 0.66–1.00), predictive positive value of 0.50 (IC 95% 0.21–1.00) and predictive negative value of 0.99 (IC 95% 0.97–1.00) (Table 3).

### Comparison of the Levels of PCT in the “cordPCT” Group vs. “delPCT” Group

The threshold values of “cordPCT” group and “delPCT” group were similar (respectively, 1.00 and 0.98 ng/mL). Their diagnostic values are given in Table 3.

**Figure 2:**
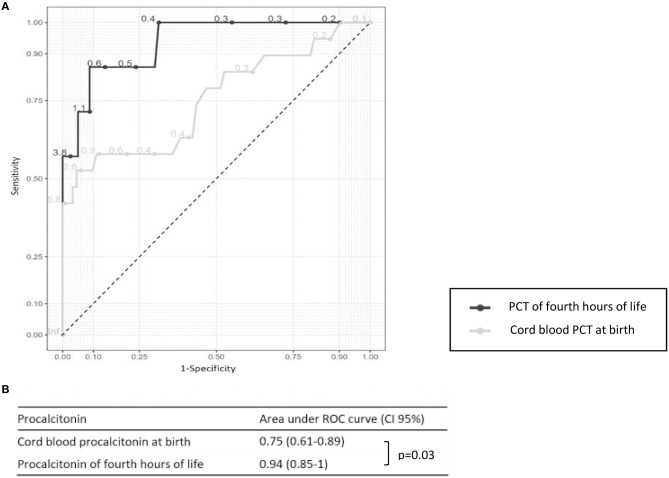
**(A)** Receiver operating characteristic (ROC) curves of cord blood procalcitonin (PCT) and PCT of 4th h of life for the whole population. **(B)** Areas under the ROC curve of cord blood PCT and PCT of the first few hours of life for the whole population with a confident interval of 95% (CI 95%).

The AUC for PCT levels obtained during the first 6 h of life were significantly higher than those of cord blood PCT; 0.94 (IC 95% 0.85–1.00) vs. 0.75 (IC 95% 0.61–0.89; *p* = 0.03) (Figure 2).

## Discussion

The main result of this study shows that the positive threshold value for PCT in a population of <32 weeks newborns was higher than that usually reported in the literature (17). One study reported a higher basal PCT level in preterm neonates compared to term neonates irrespective of the presence of infection (25). Several explanations can be given: possible association of PCT, the precursor of calcitonin with osteogenesis and metabolism of calcium (25) and frequent respiratory distress and hypoxemia in premature neonates, which are factors that increase the secretion of PCT (26, 33). The higher level of PCT reported in our study may be explained the selection of newborn with infection risk factors: 55% had prolonged membrane rupture, 49.2% was born in context of clinical chorioamnionitis, 85% presented a threat of preterm birth and 52.7% had a positive vaginal swab. This infection and/or inflammation of the intra-uterine results in an increase in the level of pro-inflammatory cytokines, such as interleukin-6 (IL-6) in the plasma of the fetus, which defines the syndrome of fetal inflammatory response (34). This pro-inflammatory cytokine along with interleukin-1β, Tumor Necrosis Factor α and endotoxines stimulate the production of PCT by the fetus (35).

However, the specificity and negative predictive value were excellent, allowing diagnosis of non-infected neonates and thus limiting prescription of antibiotics. As for the majority of studies, the sensitivity and the positive predictive value for PCT was not as good, which does not allow its use as a positive diagnostic tool (17, 36) and can be explained by a non-specific increase in PCT within a non-infectious context of asphyxia or gestational diabetes (19).

Studies concerning PCT have selected non-homogeneous populations (gestational age, number and criteria of inclusion) making difficult the comparison of our study with those of the literature. Chiesa et al. determined the value of cord PCT at 1 ng/mL for 134 neonates of an average of 33.8 weeks (31). Joram et al. obtained a threshold value of 0.6 ng/mL for a population of 812 premature neonates presenting with infection risk factors, in which 300 were <32 weeks (36.9%) (17). A recent study found a cord blood PCT threshold value of 0.7 ng/mL in a cohort of 186 extremely preterm neonates (sensitivity of 69% and specificity of 70%) and showed that premature neonates of <28 weeks may have a cord PCT threshold value higher than that used previously (24). Several studies focusing more on premature newborns remain contradictory: PCT higher, faster and longer than in the newborn at term (21) or on the contrary rather lower (22). However, our population is more premature than in these studies, which makes it original.

Further studies are required to determine the specific threshold value of premature neonates, a population at a high risk of EOS.

The physiological increase in PCT during the early days of life naturally led the majority of studies to concentrate on cord blood PCT. The urgent care of neonates in the delivery room means that the level of PCT is not always determined at birth, for example due to coagulated, insufficient or impossible to obtain cord samples. The neonatologists question the interpretation of PCT levels evaluated from samples obtained a few hours after birth due to its postnatal physiological increase. In our study the PCT threshold value in the ≪ delPCT ≫ group was similar to that of the ≪ cordPCT ≫ group (0.98 and 1.00 ng/mL, respectively). The specificity and negative predictive value of PCT in the 1st h of life remained excellent and delayed sampling at the peak plasma level during immediate postnatal infection may explain the increase in the AUC and its sensitivity. In our study samples were obtained at a maximum of 219 min of life. So it is not possible to extrapolate further to sampling between the fourth and 6th h of life. Our study is the first to our knowledge, to investigate the use in clinical practice of PCT levels sampled within 4 h of life.

This work has some limitations. Firstly the methodology used (retrospective, neonates with infection risk factors) may explain the higher PCT threshold values of our population compared to those of the literature and the high frequency of infection. Another limit is the low absolute number of proven infections that can modify the threshold established, which makes these results difficult to generalize for the moment. The low number of extreme premature newborns (<28 GA) also limited us in the interpretation of the results of this subgroup.

To confirm our findings, it is necessary to consider longitudinal prospective studies in order to define, as is the case in the surveillance of neonatal jaundice, thresholds according to the term of birth.

In conclusion, this study reports a threshold value of PCT that seems to be higher in a population of premature neonates of <32 weeks compared to full term neonates. It shows that the PCT level evaluated during the first 4 h of life was an appropriate and reliable tool in the screening for EOS.

What is already known on this topic?

- The PCT cord test is widely used in the decision algorithm for the management of neonatal early onset sepsis
- Neonatal early onset sepsis remain responsible for significant morbidity and mortality in newborns, especially premature infants
- Limiting neonatal antibiotics exposure requires effective and safe screening strategies

What this study adds?

- A better definition of the positivity threshold of the PCT assay in the very preterm newborn
- The possibility of using and interpreting the dosage of PCT not taken from the cord, up to 4 h after birth
- The need to continue the studies in a broader way to refine the interpretation according to the term of birth.

## Data Availability Statement

The original contributions generated for this study are included in the article/supplementary material, further inquiries can be directed to the corresponding author/s.

## Ethics Statement

The studies involving human participants were reviewed and approved by a favorable opinion from the ethics committee (CERNI): Approval number: 2020-67. Written informed consent for participation was not provided by the participants' legal guardians/next of kin because: Retrospective data study.

## Author Contributions

BB had contributed to the conception, the design of the work, the acquisition, analysis, interpretation of data, and drafted the work. SEDT and MM had contributed to the conception, the design of the work, and interpretation of data, and revising it critically for important intellectual content. BF-G, MB, and FC had revised it critically for important intellectual content. CD had contributed at the analysis of data for the work. All the authors were agree to be accountable for all aspects of the work in ensuring that questions related to the accuracy or integrity of any part of the work were appropriately investigated and resolved.

## Conflict of Interest

The authors declare that the research was conducted in the absence of any commercial or financial relationships that could be construed as a potential conflict of interest.

## Abbreviations

AUC: Area under the curve
CI 95%: 95% Confidence interval
CRP: C-reactive protein
EOS: Early-onset sepsis
IQR: Interquartile range
PCT: Procalcitonin
ROC: Receiver operating characteristics.
